# Supplementary material for: Predictive remapping and allocentric coding as consequences of energy efficiency in recurrent neural network models of active vision
Source: Patterns (N Y). 2025 Nov 20;7(1):101422. doi: 10.1016/j.patter.2025.101422 (PMC12827687; doi:10.1016/j.patter.2025.101422)
Supplement: Document S1. Figures S1 and S2 and supplemental methods [file mmc1.pdf]

Patterns, Volume *7*

Supplemental information

Predictive remapping and allocentric coding as consequences of energy efficiency in recurrent neural network models of active vision

Thomas Nortmann, Philip Sulewski, and Tim C. Kietzmann

Supplemental methods

ALTERNATIVE ARCHITECTURES AND OBJECTIVES

To assess the robustness and specificity of our findings to the architectural and task details, we conducted two complementary analyses. First, we evaluated how architectural variations affect the emergence of energy-efficient fixation patterns and allocentric coding by testing models with different numbers of time steps per fixation and hidden layers. Second, we investigated whether similar energy efficient computational mechanisms arise under alternative training objectives by comparing our energy-minimisation approach against supervised object categorisation, temporal contrastive learning (trained for similarity with two time steps before current time step; and dissimilarity with other scene fixations), and an untrained model. These comparisons allow us to determine whether the observed phenomena are specific to energy-efficiency constraints or represent more general properties of recurrent networks processing sequential visual input. The results demonstrate that energy-efficient fixation patterns and allocentric coding are robust across architectural variants but emerge specifically from the energy-minimisation objective rather than from alternative training regimes or network architecture alone (Supplementary Fig S1).

1. ALLOCENTRIC UNIT CLUSTERING ANALYSIS

To characterise the functional organisation of allocentric coding units, we performed k-means clustering on standardised unit activations. Unit activations were first transformed using PCA (retaining the first 10 components), with the optimal cluster number (k=7) determined by silhouette score maximisation. Representative units were identified as those most correlated with cluster centroids. Seven distinct clusters emerged from the top 22 allocentric units (11 most predictive for x and y coordinates each), revealing heterogeneous spatial selectivity patterns. The majority of units (n=15) belonged to a single large cluster, while smaller specialised clusters (n=1-3) showed distinct spatial tuning characteristics. Spatial coding patterns included selectivity for upper, lower, left, and right scene regions, as well as centre/periphery coding, suggesting functional organisation into subpopulations with largely complementary spatial coverage that supports predictive remapping (Supplementary Fig S2).

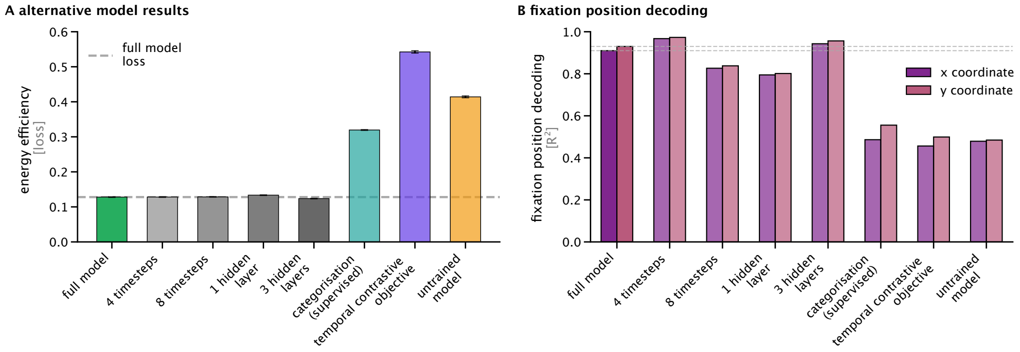

Fig. S1. Robustness of energy efficiency and allocentric coding across network architectures and objectives. **(A)** Energy efficiency loss comparison showing the full model and architectural variants: 4 and 8 time steps per fixation, 1 and 3 hidden layers, supervised categorisation training, temporal contrastive learning, and untrained baseline. All alternative architectures of the energy-minimisation model achieve similar efficiency (losses 0.124 to 0.134), while supervised training on the alternative objective of predicting the scene objects shows reduced energy efficiency (loss = 0.320, 99% CI: [0.318, 0.321]). Temporal contrastive learning as a scene specific temporal stability objective performs poorly (loss = 0.542, 99% CI: [0.539, 0.546]), worse than both trained models and the untrained baseline (loss = 0.414, 99% CI: [0.411, 0.417]). Error bars represent 99% confidence intervals computed across test set fixations. **(B)** Allocentric fixation position decoding performance across architectures. All alternative architectures of the energy-minimisation model successfully decode allocentric coordinates ($R^2 > 0.79$), while the supervised object detection model shows degraded allocentric coding ($R^2 = 0.49/0.56$). Temporal contrastive learning ($R^2 = 0.46/0.50$) performs similarly poorly. Both alternative objectives only allow for similar decoding as the completely untrained model ($R^2 = 0.48/0.48$). Dashed lines indicate full model performance for reference.

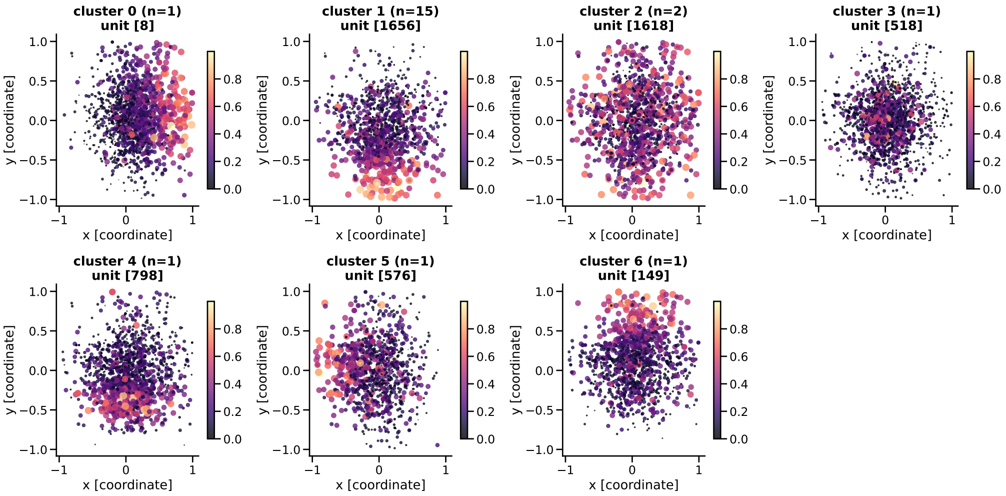

Fig. S2. Cluster representatives of allocentric coding units reveal distinct spatial tuning patterns. Spatial tuning profiles of representative units from 7 clusters identified through k-means clustering of the top 22 allocentric units (11 most predictive for x and y coordinates each). Representative units were selected as those most similar to cluster centroids. Color intensity and point size indicate normalized activation levels across spatial coordinates; magma colormap represents activation from low (dark) to high (bright yellow). Data subsampled to 4000 spatial locations for visualization. Cluster 1 contains the majority of units (n=15), while other clusters show more specialized spatial selectivity patterns. The heterogeneous tuning profiles demonstrate functional organization within the allocentric reference frame that supports predictive remapping.
