# Supplementary material for: Predictive remapping and allocentric coding as consequences of energy efficiency in recurrent neural network models of active vision
Source: Patterns (N Y). 2025 Nov 20;7(1):101422. doi: 10.1016/j.patter.2025.101422 (PMC12827687; doi:10.1016/j.patter.2025.101422)
Supplement: Document S2. Article plus supplemental information [file mmc2.pdf]

Patterns

Article

Predictive remapping and allocentric coding as consequences of energy efficiency in recurrent neural network models of active vision

Highlights

- Neural networks are optimized for energy efficiency during active vision
- Predictive remapping emerges as a consequence of energy efficiency
- An allocentric reference frame emerges that guides predictions

Authors

Thomas Nortmann, Philip Sulewski, Tim C. Kietzmann

Correspondence

tnortmann@uni-osnabrueck.de

In brief

This study gives an example of how complex computations in neural networks can emerge from simple physical principles. Training a model to optimize internal energy efficiency while performing eye movements suffices for predictive remapping to emerge. The model learns to translate eye movements into an allocentric reference frame. Based on this reference frame, it learns to predict and inhibit the next fixation.

Nortmann et al., 2026, Patterns *7*, 101422
January 9, 2026 © 2025 The Author(s). Published by Elsevier Inc.
https://doi.org/10.1016/j.patter.2025.101422

CellPress

Patterns

CellPress
OPEN ACCESS

Article

Predictive remapping and allocentric coding as consequences of energy efficiency in recurrent neural network models of active vision

Thomas Nortmann,[1,3,4,*] Philip Sulewski,[1,2,3] and Tim C. Kietzmann[1]
[1]Institute of Cognitive Science, University of Osnabrück, 49090 Osnabrück, Germany
[2]Vision and Computational Cognition Group, Max Planck Institute for Human Cognitive and Brain Sciences, 04103 Leipzig, Germany
[3]These authors contributed equally
[4]Lead contact
*Correspondence: tnortmann@uni-osnabrueck.de
https://doi.org/10.1016/j.patter.2025.101422

THE BIGGER PICTURE We show how some of the brain's amazing abilities, such as keeping our view of the world stable even when our eyes move, might come from simple ideas such as saving energy. Instead of assuming that the brain is wired with complex instructions for predicting what we will see next, we explored whether these skills could develop naturally from basic physical principles. We apply a computer model that mimics how our eyes move and how the brain processes visual information. This model was trained to perform eye movements while trying to use as little energy as possible by reducing unnecessary neural activity. Surprisingly, as the model learned to be more energy efficient, it started to develop a process called predictive remapping. This process is how the brain predicts what will appear in our vision after an eye movement, so our perception stays smooth and stable. Moreover, the model learned to create an internal representation that translates the position of the eyes into a more stable, environment-centered frame of reference. This internal map helps the system predict future visual input and decide when to inhibit or reduce certain signals, making the whole process more efficient. Altogether, we show that complex visual functions such as predictive remapping and creating an environment-centered reference frame can emerge naturally when a system is optimized for energy efficiency.

SUMMARY

Despite moving our eyes from one location to another, our perception of the world is stable—an aspect thought to rely on predictive computations that use efference copies to predict the upcoming foveal input. Are these complex computations and required connectivity scaffolds genetically encoded, or could they emerge from simpler principles? Here, we consider the organism's limited energy budget as a potential origin. We expose a recurrent neural network to sequences of fixation patches and saccadic efference copies, training the model to minimize energy consumption (preactivation). We show that targeted inhibitory predictive remapping emerges from this energy-efficiency optimization alone. Furthermore, this computation relies on the model's learned ability to re-code egocentric eye coordinates into an allocentric (image-centric) reference frame. Together, our findings suggest that both allocentric coding and predictive remapping can emerge from energy-efficiency constraints, demonstrating how complex neural computations can arise from simple physical principles.

INTRODUCTION

Although our eyes shift fixation locations approximately three times per second, we perceive the world as stable and continuous—a surprising phenomenon given the substantial changes in visual input that occur across saccadic eye movements. This "hard binding problem"[1] represents a fundamental challenge in vision science. To address it, predictions of foveal input from upcoming eye movements are thought to play a crucial role through predictive remapping.[2] These predictions rely on efference copies encoding planned eye movements to anticipate the visual consequences of saccadic eye movements.

 © 2025 The Author(s). Published by Elsevier Inc.
This is an open access article under the CC BY license (http://creativecommons.org/licenses/by/4.0/).

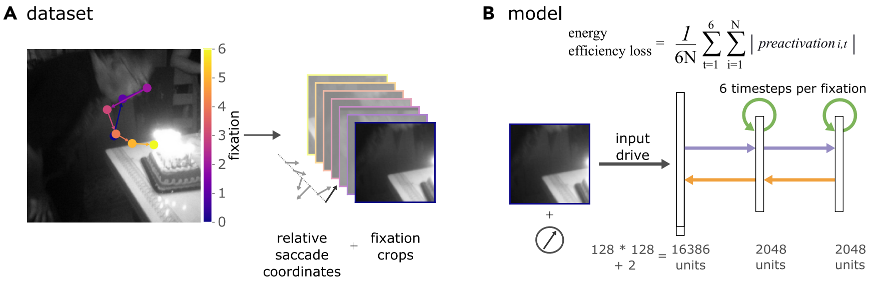

Figure 1. Minimizing preactivation in response to human-like saccade sequences

(A) Generation of fixation crop sequences with relative saccadic coordinates from the MS-COCO dataset,[28] with the sequences based on DeepGaze III.[29]

(B) Schematic of the RNN architecture alongside depiction of the energy-efficiency loss function. The model receives an excitatory input drive directly connected to the first layer (fixed, no learnable parameters) while computing through hidden layers with bottom-up (purple), lateral (green), and top-down (yellow) connections. Energy efficiency is modeled by minimizing mean absolute unit preactivation computed over all units (N) and all six time steps, which encourages sparse neural activity without excessive inhibition. The fixed excitatory input prevents the network from learning to ignore inputs. This combination aims to support the emergence of targeted inhibitory predictions rather than costly over-inhibition or network shutdown.

Behavioral evidence shows that this mechanism enables optimal transmission and integration of visual features across saccades.[3–7] A broad network of brain areas is involved in this process,[8–14] with various proposed mechanisms for remapping peripheral information to foveal locations[1,2,12,15–18] Although these remapping mechanisms are not static and can be altered through learning,[19] the fundamental computational principles underlying the emergence of such sophisticated spatial transformations remain unclear. While the exact mechanisms of predictive remapping are still a topic of debate, what remains less controversial is the presence of (pre-)saccadic predictive computations that facilitate a seamless visual handover.

The central importance of stable perception despite eye movements and the complexity of the computations involved raise intriguing questions about their origins: are these processes and underlying connectivity motifs genetically coded, or could they emerge from simpler principles? Energy efficiency stands as a fundamental constraint in neural computation and a cornerstone principle in computational neuroscience.[20,21] This principle manifests across multiple neural phenomena, from sparse coding—where information is represented by few active neurons[22]—to predictive coding, where predictable sensory inputs are actively inhibited.[23,24] Our study explores whether the sophisticated neural mechanisms that maintain perceptual stability across saccades might similarly emerge from these basic energy constraints without requiring specialized genetic coding. Within this prediction framework, inhibitory remapping provides key computational advantages by proactively suppressing neural populations activated by predictable post-saccadic visual input. This targeted inhibition reduces metabolic costs by preventing redundant processing of expected information while allocating resources to novel features that carry behaviorally relevant information.

Previous work has demonstrated that predictive visual computations can arise in systems optimized for energy efficiency.[25] However, while Ali et al.[25] studied temporally predictable digit image sequences, the mechanism underlying predictive remapping across saccades is decisively more complex: relative spatial information about saccadic targets needs to be used to flexibly transfer information across space, e.g., from the periphery to the fovea. Moreover, our approach extends this previous work by utilizing naturalistic stimuli and mirrors the spatial stochasticity of human-like fixation behavior, providing a more ecologically valid test of whether energy efficiency can drive complex spatial computations. Previous computational models have explored remapping through pre-specified circuits[26] and explicit spatial updating mechanisms.[27] In contrast, the present approach tests whether remapping can emerge from energy-efficiency constraints alone, without requiring corresponding, predefined connectivity profiles. Hence, it remains unclear whether the intricate task of predictive remapping can similarly be found as an emergent phenomenon driven by energy efficiency, without the need for intricate architectural design.

Here, we approach this question by developing a model framework in which a recurrent neural network (RNN) is subjected to sequences of fixation patches from natural scenes alongside their corresponding (relative) saccadic efferent copies (Figure 1). The RNN's objective is to optimize its synaptic weights to minimize firing rates and synaptic transmission, approximating a substantial part of the energy costs related to neural information processing. This energy-efficiency objective is implemented by minimizing the mean absolute preactivation (the sum of inputs to a model unit before the activation function is applied) across all units and time steps while we maintain excitatory input to the network. In combination, this aims to prevent costly over-inhibition or a learned network shutdown in favor of energy efficiency.

To elucidate whether minimizing a network's energy expenditure, in the context of saccadic eye movements, can yield signatures of predictive remapping, we (1) compared the energy-efficiency loss of model predictions against several control conditions, such as predicting the average training set luminance, the average training set fixation crop, and a static repetition of the previous crop as predictor, and (2) visualized and analyzed the model-internal drive, demonstrating targeted inhibition. By performing diagnostic readouts and *in silico* lesion studies, we further reveal the network's learned strategy to implement such predictive remapping. We find that the network learned to map the sequence of relative saccade targets into an allocentric reference frame and, importantly, that the allocentric-coding units are driving the targeted prediction. Finally, we contrast the energy-efficiency-trained model against two alternative training objectives: (1) supervised object categorization and (2) InfoNCE contrastive learning[30] that promotes scene-specific temporal stability. These comparisons reveal that both alternative training regimes yield low energy efficiency and impaired allocentric fixation position coding compared to the energy-minimization objective.

CellPress
OPEN ACCESS

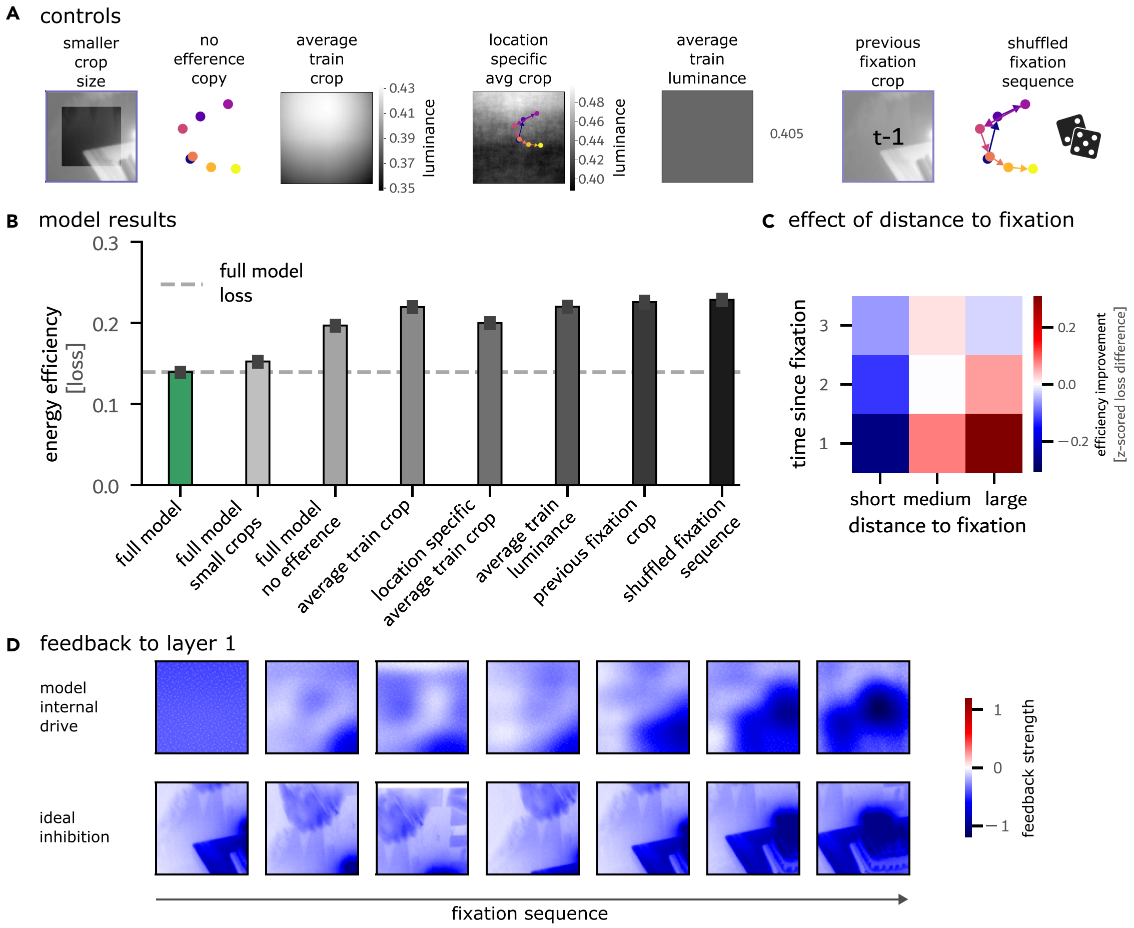

Figure 2. Inhibitory predictive remapping as a consequence of energy efficiency

(A) Illustration of the seven control conditions used for performance comparison: training with smaller crops (56% smaller), training without efference copies, location-specific average crop, average crop, average luminance value, previous fixation crop, and testing with shuffled fixation sequences.

(B) Evaluation of energy-efficiency loss in the RNN model and control conditions, highlighting the model's predictive capabilities. Error bars represent 99% confidence intervals computed across test set fixations.

(C) Mean normalized loss (Z scored by average test-set loss) plotted as a function of two variables: the spatial distance from previous fixation locations and the temporal distance (number of fixation steps) since the location was last visited.

(D) Example of the trained RNN's internal feedback to layer 1 (top row), together with the ideal inhibition defined as the inversion of the fixation crop (bottom row). Optimized for energy efficiency, targeted inhibition emerges in the trained RNN model. While smooth, the inhibitory patterns align with the ideal inhibition.

RESULTS

Inhibitory predictive remapping emerges as a consequence of energy efficiency

We trained an RNN architecture with two hidden layers to minimize its energy consumption, implemented as minimizing unit preactivation[25] while being presented with excitatory sensory input (sequences of fixation crops) and the corresponding relative saccade coordinates. Fixation sequences were generated on natural scenes sourced from the MS-COCO dataset[28] (see Figure 1A) using DeepGaze III predictions of human fixation behavior.[29] Each sequence consisted of seven fixation crops, and each crop was processed for six model time steps (Figure 1B). In our analyses, we primarily focus on the computations/loss values recorded at the first model time step of each fixation crop. This time point enables us to investigate the model's behavior while transitioning from one fixation location to the next.

To assess the performance of our model, we compared its energy consumption against a series of controls. First, we contrasted the energy-efficiency-trained RNN against static predictions derived from the training set (see Figure 2A). In one variant, the control model was set to subtract the average luminance of all images in the training set to test whether the model learned constant-value subtraction rather than spatially specific predictions. We find the loss of the energy-optimized RNN to be significantly lower ($p < 0.001$; see Figure 2B). The second static control used the average crop, computed over all crops in the training set, as an inhibitory prediction to evaluate whether the model outperforms generic inhibitory templates. Again, our RNN model loss was significantly smaller ($p < 0.001$; see Figure 2B). An additional non-static control to probe spatial biases in the dataset used the crop at the current fixation location of the average image in the training set to control for location-specific dataset biases (Figure 2A). Our RNN model loss was significantly lower

than this control condition ($p < 0.001$; see Figure 2B). These controls demonstrate that the model outperforms non-informative predictions and is sensitive to scene-specific characteristics.

Next, we asked to what extent the fixation dynamics on the scene informed the energy-efficiency-based predictions. To this end, we contrasted our model's predictions against two scene-specific control conditions (Figure 2A). First, we tested against a model that performs inhibitions based on a repetition of the last fixation crop to test whether the model merely repeats previous input rather than integrating efference copies. Our RNN model loss was lower than that of the previous fixation control ($p < 0.001$; see Figure 2B), indicating that the RNN learned to adapt its predictions in response to upcoming saccadic targets on a given scene. Second, we ran a control in which we produced a mismatch between the efference copies given to the model and the corresponding change in visual information that results from the saccade to test whether the model relies on accurate spatial information coupling. To do so, the sequences of fixation coordinates were shuffled while maintaining the original fixation crop sequence. Subsequent to this coordinate shuffling, we recomputed the relative saccade positions to be fed to the model. Our RNN model loss that relied on the original sequence of efference copies was lower than in the control condition with shuffled position-crop pairs ($p < 0.001$; see Figure 2B). This suggests that the RNN successfully couples efference-copy information to visual information at saccade targets—a strong indication that it performs inhibitory predictive remapping. To probe whether our model architecture could learn similar predictions in the absence of spatial information, we trained the same model without concatenating the efference copies to the input (Figure 2A). Our RNN model loss was lower than in this control model ($p < 0.001$; Figure 2B). This indicates that the models need to rely on efference copies to achieve good spatial predictions. To investigate the model's reliance on the overlap of fixation crops, we implemented a control model with the same architecture except for a visual field reduced by 56% to test whether predictions depend on overlapping visual content (Figure 2A). Despite this control model having a larger energy-efficiency loss than our model, it outperformed all controls (all $p < 0.001$; Figure 2B), indicating that the learned predictions do not solely rely on fixation overlap. These results are robust across architectural variations, including different numbers of hidden layers and time steps per fixation (Figure S1A). To explore whether alternative objectives might drive similarly efficient computations, we trained networks with two control objectives. First, we implemented an MS-COCO multi-hot object classification task using binary cross-entropy loss (BCELoss) on all objects per scene. Second, we used InfoNCE contrastive learning, with positive pairs being the current fixation patch paired with patches from 2 time steps back (spanning fixation transitions) and negative pairs consisting of 8 random crops from different scenes to promote scene-specific temporal stability. Both approaches resulted in less energy-efficient solutions and did not develop allocentric coordinate decoding (Figure S1).

Probing the spatial memory abilities of our model, we analyzed whether the energy-efficiency loss changes for fixations if a spatially close location was fixated earlier. Relative to the mean across fixations, the loss decreased in cases where one of the last two fixations was close to the current fixation and increased if previous fixations were far away (Figure 2C). This effect faded out toward three or more fixations in the past when in combination with large saccadic distances. However, it holds not only for the last but also the second-to-last fixation. With this, a limited form of spatial memory by the RNN is suggested.

In analyzing the model more closely, we observe that, to achieve energy efficiency in the face of excitatory input, the overall top-down feedback to layer 1 of our trained RNN was inhibitory (feedback over test-set crops: $\mu = -0.39$, 99% confidence interval [CI] = [-0.78, -0.09], $n = 86{,}142$). To better understand the spatial specificity of this model behavior, we next visualized the model-internal drive across sequences of eye movements. Visual inspection of these inhibitory signals suggests that top-down feedback is spatially specific to the saccadic target locations rather than being a global, spatially unspecific signal (Figure 2D). While smooth in nature, inhibitory predictive remapping is clearly evident in model-internal dynamics and aligns with the ideal inhibition patterns (extracted from ground-truth data; Figures 3F and 3G for a quantification). It should be noted that the predictions here are computed on held-out test images and are thus not based on memorization of a given scene. Rather, inhibition is dynamically targeted in response to efference-copy signals.

In conclusion, our RNN outperforms all controls in terms of energy efficiency and exhibits targeted inhibition in a scene-specific manner that is based on efference-copy information.

Allocentric coding emerges from energy efficiency by integrating over sequences of egocentric efference copies

While targeted predictions could be generated in a relative coding scheme, that is, generating predictions from peripheral information alone, this approach is limited by the steeply decreasing accuracy of the visual periphery. Further, given the existence of refixations, a potentially more efficient solution involves path integration of egocentric saccade commands into an allocentric coordinate system that remains stable across saccades. We therefore hypothesized that energy-efficient predictive remapping would drive the emergence of allocentric spatial coding. To test for this possibility, we trained a linear diagnostic readout to decode the allocentric fixation coordinates from the unit activations in the two hidden RNN layers. This revealed that, indeed, RNN training for energy efficiency led the model to re-code the relative efference-copy information into an allocentric code (x coordinate decoding: $R^2 = 0.91$ and y coordinate decoding: $R^2 = 0.93$; see Figures 3A and 3B). Notably, the successful readout relied on only a fraction of all units, as revealed by an analysis of the regression weights (Figure 3C). The high accuracy of the allocentric readout suggests that the model indeed constructed a stable allocentric reference frame based on egocentric efferent copies of previous fixation locations. Importantly, this allocentric-coding capacity is preserved across various architectural configurations but is absent in models trained with alternative objectives, such as supervised categorization or temporal contrastive learning (Figure S1B).

CellPress
OPEN ACCESS

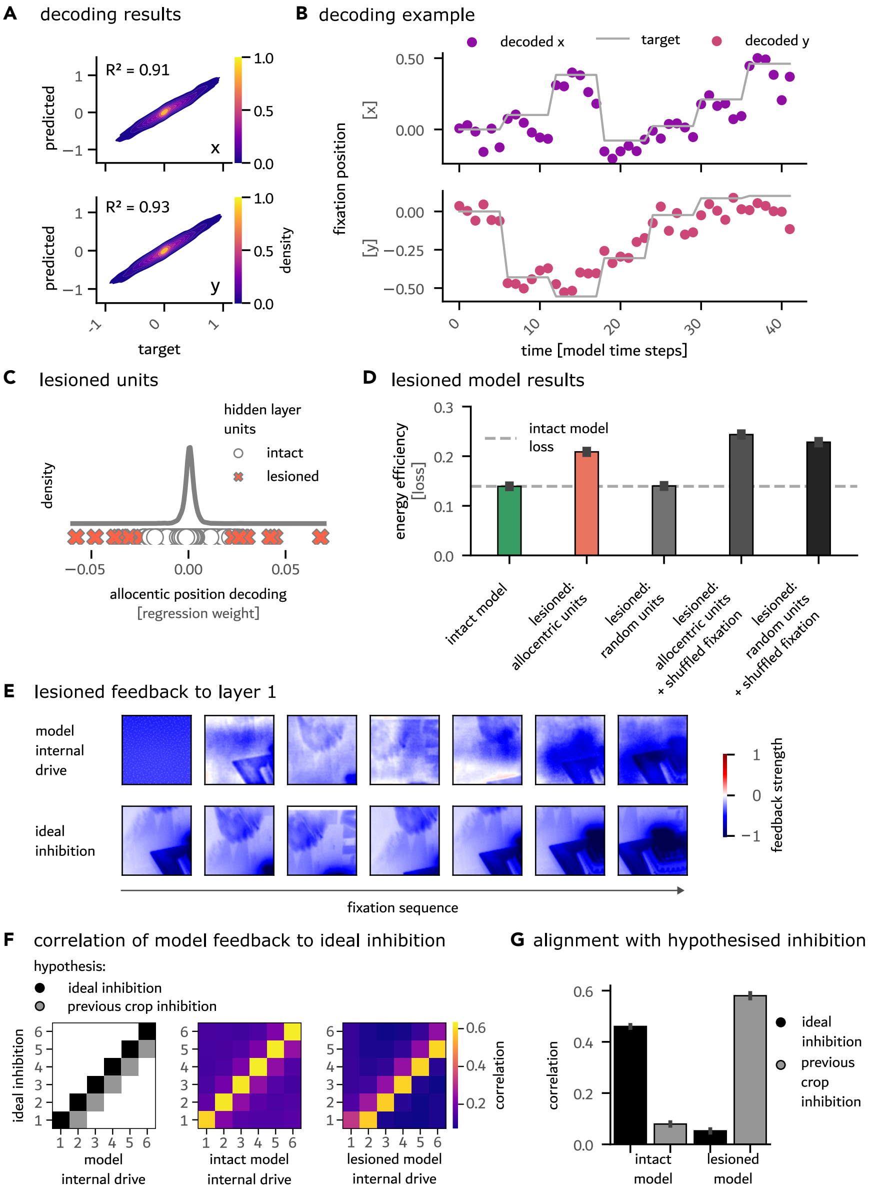

Figure 3. Emergence of an allocentric reference frame that underlies targeted predictive inhibitory remapping

(A) Linear decoding of the allocentric fixation *x* (top) and *y* (bottom) coordinates from the RNN hidden layers. The model integrates the relative egocentric saccade commands into an allocentric reference frame.

(B) Example fixation trajectory (7 fixations with 6 RNN time steps each) and the decoded coordinates.

(C) Units targeted during *in silico* lesioning were chosen based on their contribution to allocentric coordinate decoding; 0.5% of units with the highest betas were selected.

(D) Lesioning allocentric units led to a significant increase in energy consumption, the extent of which is not matched by random lesioning. Error bars represent 99% confidence intervals computed across test set fixations.

(E) Example of altered feedback to layer 1 following the lesioning of allocentric-coding units.

(F) Correlation of the intact and lesioned model feedback with the ideal inhibition targets.

(G) Correlation of matrices for observed and hypothesized prediction patterns. The internal drive of the lesioned model aligns with the current crop, whereas the intact model aligns with the ideal prediction of the future input.

Allocentric reference frame units operate as prediction units

To test for the computational role of the units coding an allocentric reference frame, we performed an *in silico* lesioning study on the trained model units. Due to the relatively small number of allocentric-coding units, we only lesioned 0.5% of all units (22 of 4,096) by setting them to zero during runtime (see Figure 3C). As a control to this targeted lesioning experiment, we randomly selected the same number of units in the network for lesioning. Random lesioning did not lead to a notable change in loss compared to the intact model. However, lesioning the units that coded for the allocentric position, selected as having large weights in our diagnostic readout, led to a significant increase in the energy-efficiency loss ($p < 0.001$; see Figure 3D). The lesioned model can no longer outperform the control model trained without efference copies ($p > 0.999$). Together, these findings demonstrate the importance of the allocentric-coding units for the model's ability to perform targeted inhibitory predictive remapping. The computational importance of the allocentric units is further underlined by the allocentric-coding units selected for lesioning having a significantly larger activity on the test set than the average hidden unit ($p < 0.001$). This heightened activity despite the training for energy efficiency serves as evidence for the functional importance of these units in minimizing energy expenditure.

To better understand how the *in silico* lesions affect network computations, we visualized the model-internal drive (Figure 3E) and compared it to the ground-truth ideal inhibition. We observe that the lesioned model reused the current crop as an inhibitory signal, in contrast to the intact model, which performed a targeted inhibition according to the expected future visual input that results from the saccade to a new location (Figure 2D). To quantify this observation, we compared the model-internal drive of both the intact and lesioned models to the ideal inhibitory signals across all time steps (correlation used as a similarity measure). We then compared the resulting similarity matrices to two predictions: one representing a case in which the current crop is used as an inhibitory signal and one where the prediction is aligned with the ideal inhibition (see Figures 3F and 3G). The model-internal inhibitory drive of the intact model aligned well with the ideal future inhibition ($r = 0.46$, $p < 0.001$). In contrast, following the targeted lesioning of the allocentric reference frame units, the model fell back to an internal dynamic in which inhibitions were aligned with the current

crop ($r = 0.58$, $p < 0.001$). Only a minimal alignment with the ideal inhibition remains after the lesioning ($r = 0.05$, $p < 0.001$). This analysis demonstrates that the intact model relied on the allocentric units to perform targeted inhibition of the expected input that results from the saccade to a new location.

To further characterize the functional organization of the allocentric-coding units, we performed a clustering analysis on their unit activation profiles, revealing seven distinct clusters with heterogeneous spatial selectivity patterns (Figure S2). Most of the units ($n = 15$) belonged to a larger group, while smaller groups ($n = 1$–3) showed additional specialized spatial-tuning characteristics. Spatial coding for upper, lower, left, and right scene regions, as well as a center/periphery coding, suggests a functional organization into subpopulations with largely complementary spatial coverage.

DISCUSSION

Energy efficiency and allocentric coding

Energy efficiency drives predictive remapping and allocentric coding

We investigated the potential origins of predictive remapping across human-like saccade sequences. Using an RNN model system, we observed that targeted inhibitory predictive remapping emerged naturally from optimizing model computations for energy efficiency. Our diagnostic readout analyses and lesion studies revealed that the model, in an effort to save energy, learned to code in an allocentric reference frame based on the relative efference copies provided to it and used the corresponding allocentric-coding units for targeted inhibition. Only a small number of units were found to be coding for allocentric positioning, consistent with sparse coding principles and with previous results that found few prediction units driving world-model-based inhibition in temporal sequence data.[25] The sparse population of allocentric units in our model (<1% of the total) requires persistent unit activity to preserve spatial reference stability. Inhibiting this persistent coding would compromise the model's remapping capacity. While this persistent coding represents an additional computational investment, it ultimately promises energy savings. Therefore, limiting the allocentric coding to a minimal fraction of units is preferable.

Allocentric coding as for perceptual stability

The emergence of units capable of translating relative to allocentric coding has significant implications for both predictive remapping and spatial memory encoding. Re-coding spatial information in allocentric terms provides the system with a stable reference frame despite constantly changing fixation locations, enabling object information to be encoded consistently. Such a code may not only underlie our stable perception of the world but may also serve as spatial memory of where things are in our surroundings, enabling the system to use the world itself as an external memory for detailed visual information not maintained in working memory.[31,32]

Emergence of complex computation from simple principles

The emergence of allocentric units in an unsupervised manner—as a result of energy-efficient coding—is particularly noteworthy. Previous work studying re-coding from relative to allocentric reference frames in path integration tasks explicitly trained supervised RNNs to perform this mapping.[33] In such supervised approaches, an open question remained regarding how the necessary training data could be obtained by a biological system. Our work suggests that unsupervised objectives, such as energy efficiency, may be sufficient to yield similar computations without requiring explicit supervision signals. This finding supports the broader principle that complex computational mechanisms can emerge from simple constraints. Rather than requiring genetically coded circuits specifically designed for predictive remapping, our results indicate that fundamental physical constraints—such as the brain's limited energy budget—can drive the emergence of sophisticated computational solutions to complex perceptual problems.

Shared coding principles for vision and navigation

The emergence of a population of units that allows for the readout of allocentric coordinates, as seen in our model, suggests that an active visual system can use this coding scheme to be energy efficient. The existence of allocentric coding does not, however, imply that the corresponding neurons would have to reside within the visual system. For example, spatial coding schemes are commonly observed in the hippocampal-entorhinal circuits, which also code for eye-movement locations in visual space.[34,35] Accordingly, the hippocampal formation may employ similar mechanisms to code for world-centered visual space as it does for navigable space. Additionally, similar to spatial navigation, the hippocampal formation likely interacts with regions such as the posterior parietal cortex (PPC) and retrosplenial cortex (RSC) to convert egocentric viewing coordinates into allocentric ones.[36,37] Our computational model provides a mechanistic basis for understanding how these transformations might occur and demonstrates that such sophisticated spatial coding can emerge through energy optimization processes alone, without requiring pre-specified spatial mapping circuits.

Nature of predictive signals and biological plausibility

The model's predictive signals exhibit spatially targeted but coarse-grained characteristics, with smooth inhibitory patterns that broadly suppress expected input locations rather than precisely matching detailed visual features (Figures 2D and 3E). The energy-efficiency objective seemingly drives this spatial profile by favoring broad suppression over costly, detailed inhibition. This could be due to the model's architecture: our simple RNN architecture, which includes fully connected layers, likely lacks the multi-leveled retinotopic organization present across visual areas in the brain, thereby likely contributing to the observed smooth spatial characteristics. At the same time, it is conceivable that the targeted, albeit coarse-grained, inhibition aligns with spatial pointer theories of remapping,[1,10,38] which propose that remapping operates through attention-based spatial markers rather than detailed feature transfer. However, the inhibitory nature of the modulations observed in our model differs from the typically excitatory attention mechanisms suggested in pointer theories. This seeming misalignment between theories opens interesting avenues for future research that could investigate how attentional pointers and targeted inhibition could interact during active vision.

Outlook and future research

Integrated active vision and navigation frameworks

Future work could focus on integrating our model into a larger framework of active vision involving working memory and task-guided computations. Such a comprehensive framework would allow us to study further computational benefits of emergent allocentric coding, including targeted information integration, object localization, and more efficient memory encoding. This extended framework could provide insights into the functional separation among multiple brain regions involved in saccadic remapping, including the superior colliculus, frontal eye fields, entorhinal cortex, and various visual cortical areas.[8,35,39–43] Such a more elaborate model architecture might also result in more expressive spatial memory abilities than observed in this simple RNN architecture. A particularly promising direction is exploring the relationship between our findings and grid-coding mechanisms found in both spatial navigation[44–46] and primate active vision.[34,35] Our model could be extended to investigate factors that yield explicit grid-coding schemes supporting allocentric coding in visual attention, potentially bridging the gap between navigation research and visual perception studies.

Biological constraints on energy and sampling

Further investigation into more biologically realistic modeling approaches promises to be beneficial. While minimizing mean absolute preactivation is a useful proxy encouraging sparse activations and reducing costly over-inhibition, it remains an imperfect measure, as it does not exhaustively capture the complex metabolic processes underlying neural activity.[25] We acknowledge that while energy efficiency is conceptually simple, our implementation relies on backpropagation through time (BPTT). This learning rule is arguably of limited biological plausibility (see, e.g., Lillicrap and Santoro[47]). Future work should explore more biologically realistic learning rules that allow approximating the energy minimization in a more direct fashion. Similarly, incorporating retina-like sampling methods (see, e.g., Lukanov et al.[48]) would move our model closer to biological reality. This enhancement could provide a more realistic visual periphery to better predict forthcoming visual input and mirror the computational challenge faced by the visual system in translating peripheral neural representations into foveal ones (e.g., by accounting for foveal magnification).

Distributed information transfer mechanisms

It will be valuable to study how the implicit world model in our RNN, necessary for allocentric coding and targeted inhibition, is represented in this neuroconnectionist system.[49] Understanding how information can be transferred from one topographical location to another—required for pre-saccadic predictions based on peripheral views—would illuminate the recurrent computations spanning hierarchical levels of abstraction.

Neuroscientific implications

The modeling work at hand prompts several testable predictions: (1) a small subset of neurons should be dedicated to allocentric spatial coding, with a disproportionate impact on visual stability if selectively manipulated; (2) neural mechanisms supporting spatial navigation and visual stability should overlap significantly, predicting that patients with hippocampal-entorhinal-retrosplenial damage would show specific deficits in maintaining visual stability across multiple saccades; and (3) disrupting allocentric-coding mechanisms should selectively impair predictive remapping while preserving basic visual processing. These predictions offer a framework for empirical validation of our model's core premise that energy constraints naturally drive the emergence of sophisticated visual stability mechanisms.

Conclusion

In summary, our findings suggest that striving for energy efficiency as a learning objective can yield complex network behaviors, such as targeted inhibitory predictive remapping and allocentric coding of eye positions. These results contribute to a broader paradigm shift in computational neuroscience by demonstrating how fundamental physical constraints can drive the emergence of sophisticated neural computations without requiring genetically coded architectural designs. Our model thereby provides a potential solution to the hard binding problem through simple physical principles: energy-efficient networks can emergently develop mechanisms that support perceptual stability across saccades.

METHODS

Conceptual framework

The input drive is the fixed excitatory visual input provided to the model, consisting of fixation crops (gray scaled) and saccade coordinates that the network must process.

Preactivation is the sum of inputs to a model unit before the activation function is applied. This serves as a proxy for energy consumption that would occur in biological neural systems.

Energy-efficiency loss is the objective function (mean absolute preactivation across units) used to train the network. Minimizing this encourages sparse neural activity, while the fixed excitatory input prevents network shutdown, forcing the development of targeted inhibitory predictions.

The internal drive is feedback from higher to lower network layers that counteracts upcoming sensory input, quantified as the model's learned weights applied to unit activations.

The efference copy is a two-dimensional vector (Δx,Δy) modeling the saccadic movement plan as a relative positional change in pixels between fixations, provided to the model before each saccade.

Egocentric coding is a representation of positions relative to the current fixation point, implemented in our model as relative coordinate inputs that change with each new fixation.

Allocentric coding is an image-centered (rather than fixation-centered) position representation that emerges in our network, enabling consistent spatial encoding across multiple saccades despite changing viewpoints.

Dataset and task design

Naturalistic stimuli were sourced from the MS-COCO dataset,[28] with human-like fixation sequences generated via the DeepGaze III model.[29] Each input sequence included seven fixations. A training set of 48,236 images was selected, with an additional test set of 2,051 images. All reported results are based on this held-out test set of scenes never encountered during training, ensuring evaluation on unseen scenes. For each image, 10 different fixation sequences were generated. In each training epoch, one sequence with 7 fixations was randomly chosen. The fixation sequences start in the center of the scene. The

original gray-scaled scenes have a size of 256 × 256 pixels; the fixation crops were selected to be 128 × 128 pixels.

Model design

The model architecture consisted of a fully connected RNN with two hidden layers (2,048 units each) and lateral connections. For each time step t in the sequence, the input to the network was defined as

$$\mathbf{x}_t = [\mathbf{v}_t, \Delta\mathbf{p}_t]^T,$$

where $\mathbf{v}_t \in \mathbf{R}^{128\times128}$ represents the flattened visual input (fixation crop) at time t and $\Delta\mathbf{p}_t = [\Delta x_t, \Delta y_t] \in \mathbf{R}^2$ represents the relative change in fixation position in pixels (efference copy). All components of $\mathbf{v}_t$ were strictly non-negative. Analogous to Ali et al.,[25] the input to the model (input drive) was set to be identical to the input image, with no learnable parameters. This ensured that the model could not learn to ignore the input to save energy.

In contrast to Ali et al.,[25] a hierarchical architecture of the model was chosen over a reservoir of units. This change in architecture was done to allow for greater control over the number of parameters and expressivity of the network, as the size of the hidden layers can be easily adjusted to reduce computational complexity, in contrast to image size controlling a large part of the parameter count through lateral connections. The choice for a hierarchical network also aligns with previous research showing alignment between hierarchical networks and the visual cortex.[50,51]

The weight matrices were uniformly initialized following He et al.[52] with weights sampled from $\mathcal{U}\left(-\sqrt{2}\cdot\sqrt{3/N_{in}}, \sqrt{2}\cdot\sqrt{3/N_{in}}\right)$, where N_{in} is the input size of each layer and the scaling factor $\sqrt{2}$ is chosen for rectified linear unit (ReLU) activations. Bottom-up, lateral, and top-down connections operated over six time steps per fixation crop, with the last three steps adding the relative coordinates toward the upcoming fixation location (efference copy). ReLU nonlinearity was applied before computing bottom-up, lateral, and top-down connections. Before the first time step of each fixation sequence, a zero vector was passed to the model to allow for an initial inhibition of the first input.

Control models

For the control "no efference copy" (Figure 2A), the network architecture was identical except that the efference copy was not concatenated to the input, reducing the input to $\mathbf{x}_t = [\mathbf{v}_t]^T$. For the control "smaller crop size" (Figure 2A), the only change to the model design was a reduction of the visual field by 56% to 85 × 85 pixels, resulting in $\mathbf{v}_t \in \mathbf{R}^{85\times85}$.

Alternative architectures

To test the robustness of energy-efficient predictive remapping across different network configurations, we evaluated architectural variants of the base model. We trained models with reduced temporal processing (4 time steps per fixation instead of 6), extended temporal processing (8 time steps per fixation), reduced network depth (1 hidden layer instead of 2, with the hidden layer size increased to 4,096 units), and increased network depth (3 hidden layers instead of 2, with the hidden layer size decreased to 1,024 units). Paralleling the original model, the efference copy was injected after 50% of the time steps per fixation. All architectural variants maintained the same training objective (energy efficiency via L1 preactivation loss), input structure (fixation crops plus efference copies), and training parameters as the original model (Figure S1).

Training regime

Energy-efficiency loss

The RNN model was trained to minimize metabolic energy consumption, utilizing the mean absolute (L1) preactivation as a loss function. Following Ali et al.,[25] we chose preactivation as our energy proxy because it jointly captures costs from both synaptic transmission and action potentials. As theoretically derived by Ali et al., minimizing preactivation drives unit outputs toward zero (reducing firing rates) while also minimizing synaptic weight magnitudes (reducing transmission costs). Furthermore, they showed that this approach outperforms alternatives such as L1 activity or L1 activity with L2 weight penalties, encouraging sparse coding without the excessive inhibition that can result from other energy measures. The preactivation loss function is formulated as

$$\mathcal{L} = \frac{1}{6N}\sum_{t=1}^{6}\sum_{i=1}^{N}\left|\text{preactivation}_{i,t}\right|,$$

where $\mathcal{L}$ represents the energy-efficiency loss, N is the number of units, and $|\text{preactivation}_{i,t}|$ denotes the absolute preactivation value of unit i at time step t. This formulation approximates the biological energy costs associated with neural firing rates and synaptic transmission. The loss was applied across all layers to enforce energy-efficient processing.

Training was done through stochastic gradient descent with the Adam optimizer, a learning rate of 0.0005, and a batch size of 1,024. The learning rate was rescaled for each weight matrix by the size of the previous layer, as suggested by Roberts and Yaida,[53] to allow for equal contribution of all units to the learning dynamics. This results in an effective learning rate for the bottom-up connections from the first model layer (receiving image and efferent-copy data) to the first hidden layer of $lr = \frac{0.0005\cdot2{,}048}{16{,}384} = 0.125\cdot0.0005$ and an unchanged learning rate of 0.0005 for all other weight matrices. To evaluate the specificity of energy-efficient fixation patterns to the energy-minimization objective, we trained networks using two alternative objectives and compared their performance to both the original energy-minimization model and an untrained baseline (Figure S1).

Supervised object categorization

Here, the model was trained using supervised learning to predict scene category labels from MS-COCO[28] images. The supervised model used the same base RNN architecture as the energy-minimization model but with an additional output layer connecting to the second RNN layer (the final hidden layer of the model). This output layer contained 91 units with sigmoid activation functions, corresponding to the 91 object categories in the MS-COCO dataset. The model processed all 7 fixations with 6 time steps each, identical to the energy-minimization training regime. The categorical prediction was computed from the output of the final time step of the final fixation in each sequence. Training used BCELoss between the model output and the 91-class multi-hot-encoded ground-truth labels from COCO. Due to starting overfitting, the reported results are based on 150 epochs of training. This objective tests whether

energy-efficient fixations emerge from general visual processing and object recognition rather than the specific energy-minimization constraint.

Temporal contrastive learning

We implemented a temporal stability objective using contrastive learning to encourage consistent representations across temporally adjacent fixations within the same scene. The model was trained to minimize the distance between representations of consecutive fixations while maximizing distance from negative samples drawn from different scenes. The temporal contrastive model used the same base architecture as the energy-minimization model (2,048 hidden units, 6 time steps per fixation) with an InfoNCE loss[30] function using temperature parameter $\tau = 0.1$. Negative sampling used 8 fixation crops, sampled from different scenes. The positive samples were the current model time step and 2 time steps previously. This positive pair could be both within and across model fixations. Training proceeded for 150 epochs. The contrastive loss encouraged temporal stability by learning representations that remain consistent across the sequential fixation pattern within each scene while remaining distinct across different scenes.

Untrained baseline

To establish a lower bound for performance, we evaluated networks with randomly initialized weights using the same architecture as trained models without any training. This baseline isolates the contribution of the network architecture from learned representations and provides a control for comparing the effectiveness of different training objectives.

All alternative models were evaluated using identical protocols for energy efficiency (L1 preactivation loss) and allocentric position decoding as the original energy-minimization model.

Analysis

Energy-efficiency evaluation

To establish that the learned feedback to layer 1 was inhibitory, we computed the mean feedback drive over all units (128×128; see Figure 1B) per time step and per fixation crop. We then report the average and 99% CI based on the resulting distribution of means. The reported comparisons between model energy efficiency against the control conditions are based on independent t tests that are run over the loss values of all fixations in the test set ($N = 2{,}051$ scenes $\times$ 7 fixations = 14,357). For visualization (Figures 1A, 2D, and 3E), the image with the lowest model loss of all images in the test set that fulfill the criteria of having a standard deviation computed over the pixel values that exceeds 0.25 was chosen. The criterion of a high pixel standard deviation was chosen to allow for an easily visible contrast in the image. The same image and fixation path were chosen for the decoding example (Figure 3B).

To evaluate the effect of the distance of previously visited fixations to the current fixation on the energy efficiency of the network (Figure 2C), we computed the pairwise distances of all fixations of all sequences of the test set and determined the 33.3 and 66.7 percentiles as thresholds for classifying short, medium, and long distances between fixations. The losses of the second fixation of all pairs of fixations no more than 3 time steps apart were classified, given the distance and number of time steps between the fixations. The mean Z scored losses were reported.

Targeted in silico *lesioning experiments*

To determine the presence of an allocentric coordinate frame, linear decoders were fitted to the 2 normalized hidden layer activations (concatenated over both layers and all model time steps) to predict global fixation coordinates. Subsequently, the impact of specific units on model behavior was explored through a lesioning analysis. For this, the top 0.05% of units with the strongest regression coefficients for the global coordinate decoding (total: 22 units) were targeted. As a control, the same number of randomly selected units was lesioned. To quantify the impact of the targeted lesioning on both the visual precision of the inhibitory feedback and its adaptation to the fixation dynamics, we correlated the observed model-internal drive with the ideal inhibition of the upcoming crop across the full fixation sequence. The ideal inhibition pattern was computed by taking the negative of the exact upcoming fixation patch. The resulting similarity matrix describes the alignment of the model-internal drive at each time point to each of the ideal predictions. When we applied to the full test set, we computed 2,051 similarity matrices. To see whether the model's internal dynamics were related to the saccade targets (i.e., whether the model inhibited the future input) or whether the model used the current fixation patch as an inhibition template, we defined two corresponding hypothetical similarity matrices (see Figure 3F). We correlate these two hypothesis matrices with the observed similarity matrices, both for the lesioned and intact models. We quantify the fit of the hypothesis matrices by reporting the mean correlation over the test set. Statistical testing was performed against a null effect with a one-sample t test (see Figure 3G).

Allocentric unit clustering analysis

Standardized unit activations were clustered using k-means on principal-coordinate analysis (PCA)-transformed data (first 10 components), with the optimal cluster number determined (k = 7) by silhouette score maximization. Representative units were identified as those most correlated with cluster centroids. Spatial tuning was visualized (see Figure S2) using 4,000 subsampled coordinates with normalized activation levels.

RESOURCE AVAILABILITY

Lead contact

Requests for further information and resources should be directed to and will be fulfilled by the lead contact, Thomas Nortmann (tnortmann@uni-osnabrueck.de).

Materials availability

This study did not generate new materials.

Data and code availability

Our source code is available at GitHub (https://github.com/KietzmannLab/EfficientRemapping) and has been archived at Zenodo.[54] The used dataset is archived at Zenodo.[55]

ACKNOWLEDGMENTS

This work was supported by the Deutsche Forschungsgemeinschaft (DFG, German Research Foundation; 456666331). P.S. and T.C.K. were supported by the ERC Starting Grant TIME (101039524). P.S. was supported by the Federal Ministry of Education and Research (BMBF; Studienstiftung des deutschen Volkes), the Max Planck Society (MPG; Max-Planck-School of Cognition), and Deutsche Forschungsgemeinschaft (DFG; German Research

Foundation; GRK 2340). The authors thank Dr. Varun Kapoor for assisting with the dataset creation.

AUTHOR CONTRIBUTIONS

Conceptualization, T.C.K. and P.S.; data curation, T.N.; formal analysis, T.N.; funding acquisition, T.C.K.; investigation, T.N., P.S., and T.C.K.; methodology, T.N., P.S., and T.C.K.; project administration, P.S. and T.C.K.; resources: T.C.K.; software, T.N.; supervision, P.S. and T.C.K.; validation, T.N. and P.S.; visualization, T.N., P.S., and T.C.K.; writing – original draft, P.S., T.N., and T.C.K.; writing – review & editing, P.S., T.C.K., and T.N.

DECLARATION OF INTERESTS

The authors declare no competing interests.

SUPPLEMENTAL INFORMATION

Supplemental information can be found online at https://doi.org/10.1016/j.patter.2025.101422.

Received: June 5, 2025
Revised: September 8, 2025
Accepted: October 20, 2025
Published: November 20, 2025

REFERENCES

1. Cavanagh, P., Hunt, A.R., Afraz, A., and Rolfs, M. (2010). Visual stability based on remapping of attention pointers. Trends Cogn. Sci. *14*, 147–153. https://doi.org/10.1016/j.tics.2010.01.007.
2. Golomb, J.D., and Mazer, J.A. (2021). Visual Remapping. Annu. Rev. Vis. Sci. *7*, 257–277. https://doi.org/10.1146/annurev-vision-032321-100012.
3. Fabius, J.H., Fracasso, A., Acunzo, D.J., Van Der Stigchel, S., and Melcher, D. (2020). Low-Level Visual Information Is Maintained across Saccades, Allowing for a Postsaccadic Handoff between Visual Areas. J. Neurosci. *40*, 9476–9486. https://doi.org/10.1523/JNEUROSCI.1169-20.2020.
4. Ganmor, E., Landy, M.S., and Simoncelli, E.P. (2015). Near-optimal integration of orientation information across saccades. J. Vis. *15*, 8. https://doi.org/10.1167/15.16.8.
5. Harrison, W.J., Retell, J.D., Remington, R.W., and Mattingley, J.B. (2013). Visual Crowding at a Distance during Predictive Remapping. Curr. Biol. *23*, 793–798. https://doi.org/10.1016/j.cub.2013.03.050.
6. He, D., Mo, C., and Fang, F. (2017). Predictive feature remapping before saccadic eye movements. J. Vis. *17*, 14. https://doi.org/10.1167/17.5.14.
7. Wolf, C., and Schütz, A.C. (2015). Trans-saccadic integration of peripheral and foveal feature information is close to optimal. J. Vis. *15*, 1. https://doi.org/10.1167/15.16.1.
8. Duhamel, J.-R., Colby, C.L., and Goldberg, M.E. (1992). The Updating of the Representation of Visual Space in Parietal Cortex by Intended Eye Movements. Science *255*, 90–92. https://doi.org/10.1126/science.1553535.
9. Kämmer, L., Kroell, L.M., Knapen, T., Rolfs, M., and Hebart, M.N. (2025). Feedback of peripheral saccade targets to early foveal cortex. eLife *14*. https://doi.org/10.7554/eLife.107053.1.
10. Knapen, T., Swisher, J.D., Tong, F., and Cavanagh, P. (2016). Oculomotor Remapping of Visual Information to Foveal Retinotopic Cortex. Front. Syst. Neurosci. *10*, 54. https://doi.org/10.3389/fnsys.2016.00054.
11. Nakamura, K., and Colby, C.L. (2002). Updating of the visual representation in monkey striate and extrastriate cortex during saccades. Proc. Natl. Acad. Sci. USA *99*, 4026–4031. https://doi.org/10.1073/pnas.052379899.
12. Neupane, S., Guitton, D., and Pack, C.C. (2016). Two distinct types of remapping in primate cortical area V4. Nat. Commun. *7*, 10402. https://doi.org/10.1038/ncomms10402.
13. Umeno, M.M., and Goldberg, M.E. (1997). Spatial Processing in the Monkey Frontal Eye Field. I. Predictive Visual Responses. J. Neurophysiol. *78*, 1373–1383. https://doi.org/10.1152/jn.1997.78.3.1373.
14. Walker, M.F., Fitzgibbon, E.J., and Goldberg, M.E. (1995). Neurons in the monkey superior colliculus predict the visual result of impending saccadic eye movements. J. Neurophysiol. *73*, 1988–2003. https://doi.org/10.1152/jn.1995.73.5.1988.
15. Arkesteijn, K., Belopolsky, A.V., Smeets, J.B.J., and Donk, M. (2019). The Limits of Predictive Remapping of Attention Across Eye Movements. Front. Psychol. *10*, 1146. https://doi.org/10.3389/fpsyg.2019.01146.
16. Melcher, D. (2007). Predictive remapping of visual features precedes saccadic eye movements. Nat. Neurosci. *10*, 903–907. https://doi.org/10.1038/nn1917.
17. Neupane, S., Guitton, D., and Pack, C.C. (2020). Perisaccadic remapping: What? How? Why? Rev. Neurosci. *31*, 505–520. https://doi.org/10.1515/revneuro-2019-0097.
18. Rolfs, M. (2015). Attention in Active Vision: A Perspective on Perceptual Continuity Across Saccades. Perception *44*, 900–919. https://doi.org/10.1177/0301006615594965.
19. Masselink, J., and Lappe, M. (2021). Visuomotor learning from postdictive motor error. eLife *10*, e64278. https://doi.org/10.7554/eLife.64278.
20. Lennie, P. (2003). The Cost of Cortical Computation. Curr. Biol. *13*, 493–497. https://doi.org/10.1016/S0960-9822(03)00135-0.
21. Sterling, P., and Laughlin, S. (2015). Principles of Neural Design (MIT Press).
22. Olshausen, B.A., and Field, D.J. (2004). Sparse coding of sensory inputs. Curr. Opin. Neurobiol. *14*, 481–487. https://doi.org/10.1016/j.conb.2004.07.007.
23. Rao, R.P., and Ballard, D.H. (1999). Predictive coding in the visual cortex: a functional interpretation of some extra-classical receptive-field effects. Nat. Neurosci. *2*, 79–87. https://doi.org/10.1038/4580.
24. Friston, K. (2005). A theory of cortical responses. Philos. Trans. R. Soc. Lond. B Biol. Sci. *360*, 815–836. https://doi.org/10.1098/rstb.2005.1622.
25. Ali, A., Ahmad, N., De Groot, E., Johannes Van Gerven, M.A., and Kietzmann, T.C. (2022). Predictive coding is a consequence of energy efficiency in recurrent neural networks. Patterns *3*, 100639. https://doi.org/10.1016/j.patter.2022.100639.
26. Rao, H.M., Mayo, J.P., and Sommer, M.A. (2016). Circuits for presaccadic visual remapping. J. Neurophysiol. *116*, 2624–2636. https://doi.org/10.1152/jn.00182.2016.
27. Bergelt, J., and Hamker, F.H. (2019). Spatial updating of attention across eye movements: A neuro-computational approach. J. Vis. *19*, 10. https://doi.org/10.1167/19.7.10.
28. Lin, T.-Y., Maire, M., Belongie, S., Bourdev, L., Girshick, R., Hays, J., Perona, P., Ramanan, D., Zitnick, C.L., and Dollár, P. (2015). Microsoft COCO: Common Objects in Context. In European conference on computer vision (Springer International Publishing), pp. 740–755. https://doi.org/10.48550/arXiv.1405.0312.
29. Kümmerer, M., Bethge, M., and Wallis, T.S.A. (2022). DeepGaze III: Modeling free-viewing human scanpaths with deep learning. J. Vis. *22*, 7. https://doi.org/10.1167/jov.22.5.7.
30. Dave, I., Gupta, R., Rizve, M.N., and Shah, M. (2022). TCLR: Temporal contrastive learning for video representation. Comput. Vis. Image Understand. *219*, 103406. https://doi.org/10.1016/j.cviu.2022.103406.
31. O'Regan, J.K. (1992). Solving the "real" mysteries of visual perception: The world as an outside memory. Can. J. Psychol. *46*, 461–488. https://doi.org/10.1037/h0084327.
32. Somai, R.S., Schut, M.J., and Van der Stigchel, S. (2020). Evidence for the world as an external memory: A trade-off between internal and external visual memory storage. Cortex *122*, 108–114. https://doi.org/10.1016/j.cortex.2018.12.017.

CellPress
OPEN ACCESS

33. Banino, A., Barry, C., Uria, B., Blundell, C., Lillicrap, T., Mirowski, P., Pritzel, A., Chadwick, M.J., Degris, T., Modayil, J., et al. (2018). Vector-based navigation using grid-like representations in artificial agents. Nature *557*, 429–433. https://doi.org/10.1038/s41586-018-0102-6.

34. Killian, N.J., Jutras, M.J., and Buffalo, E.A. (2012). A map of visual space in the primate entorhinal cortex. Nature *491*, 761–764. https://doi.org/10.1038/nature11587.

35. Nau, M., Julian, J.B., and Doeller, C.F. (2018). How the Brain's Navigation System Shapes Our Visual Experience. Trends Cogn. Sci. *22*, 810–825. https://doi.org/10.1016/j.tics.2018.06.008.

36. Byrne, P., Becker, S., and Burgess, N. (2007). Remembering the past and imagining the future. Psychol. Rev. *114*, 340–375. https://doi.org/10.1037/0033-295X.114.2.340.

37. Clark, B.J., Simmons, C.M., Berkowitz, L.E., and Wilber, A.A. (2018). The retrosplenial-parietal network and reference frame coordination for spatial navigation. Behav. Neurosci. *132*, 416–429. https://doi.org/10.1037/bne0000260.

38. Rolfs, M., Jonikaitis, D., Deubel, H., and Cavanagh, P. (2011). Predictive remapping of attention across eye movements. Nat. Neurosci. *14*, 252–256. https://doi.org/10.1038/nn.2711.

39. Graziano, M.S.A., Taylor, C.S.R., Moore, T., and Cooke, D.F. (2002). The Cortical Control of Movement Revisited. Neuron *36*, 349–362. https://doi.org/10.1016/S0896-6273(02)01003-6.

40. Leigh, R.J., and Zee, D.S. (2015). The Neurology of Eye Movements (Oxford University Press).

41. Sommer, M.A., and Wurtz, R.H. (2006). Influence of the thalamus on spatial visual processing in frontal cortex. Nature *444*, 374–377. https://doi.org/10.1038/nature05279.

42. Sparks, D.L. (2002). The brainstem control of saccadic eye movements. Nat. Rev. Neurosci. *3*, 952–964. https://doi.org/10.1038/nrn986.

43. Wurtz, R.H., and Hikosaka, O. (1986). Role of the basal ganglia in the initiation of saccadic eye movements. In Progress in Brain Research vol. 64 of The Oculomotor and Skeletalmotor Systems: Differences and Similarities, H.J. Freund, U. Büttner, B. Cohen, and J. Noth, eds. (Elsevier), pp. 175–190. https://doi.org/10.1016/S0079-6123(08)63412-3.

44. Doeller, C.F., Barry, C., and Burgess, N. (2010). Evidence for grid cells in a human memory network. Nature *463*, 657–661. https://doi.org/10.1038/nature08704.

45. Hafting, T., Fyhn, M., Molden, S., Moser, M.-B., and Moser, E.I. (2005). Microstructure of a spatial map in the entorhinal cortex. Nature *436*, 801–806. https://doi.org/10.1038/nature03721.

46. Sargolini, F., Fyhn, M., Hafting, T., McNaughton, B.L., Witter, M.P., Moser, M.-B., and Moser, E.I. (2006). Conjunctive Representation of Position, Direction, and Velocity in Entorhinal Cortex. Science *312*, 758–762. https://doi.org/10.1126/science.1125572.

47. Lillicrap, T.P., and Santoro, A. (2019). Backpropagation through time and the brain. Curr. Opin. Neurobiol. *55*, 82–89. https://doi.org/10.1016/j.conb.2019.01.011.

48. Lukanov, H., König, P., and Pipa, G. (2021). Biologically Inspired Deep Learning Model for Efficient Foveal-Peripheral Vision. Front. Comput. Neurosci. *15*, 746204. https://doi.org/10.3389/fncom.2021.746204.

49. Doerig, A., Sommers, R.P., Seeliger, K., Richards, B., Ismael, J., Lindsay, G.W., Kording, K.P., Konkle, T., van Gerven, M.A.J., Kriegeskorte, N., and Kietzmann, T.C. (2023). The neuroconnectionist research programme. Nat. Rev. Neurosci. *24*, 431–450. https://doi.org/10.1038/s41583-023-00705-w.

50. Kietzmann, T.C., Spoerer, C.J., Sörensen, L.K.A., Cichy, R.M., Hauk, O., and Kriegeskorte, N. (2019). Recurrence is required to capture the representational dynamics of the human visual system. Proc. Natl. Acad. Sci. USA *116*, 21854–21863. https://doi.org/10.1073/pnas.1905544116.

51. Yamins, D.L.K., and DiCarlo, J.J. (2016). Using goal-driven deep learning models to understand sensory cortex. Nat. Neurosci. *19*, 356–365. https://doi.org/10.1038/nn.4244.

52. He, K., Zhang, X., Ren, S., and Sun, J. (2015). Delving Deep into Rectifiers: Surpassing Human-Level Performance on ImageNet Classification. In Proceedings of the IEEE international conference on computer vision, pp. 1026–1034.

53. Roberts, D.A., and Yaida, S. (2022). The Principles of Deep Learning Theory: An Effective Theory Approach to Understanding Neural Networks (Cambridge University Press). https://doi.org/10.1017/9781009023405.

54. Nortmann, T., Sulewski, P., and Kietzmann, T. C. Code for nortmann, sulewski & kietzmann (2025) (2025). https://doi.org/10.5281/zenodo.17214743.

55. Nortmann, T., Sulewski, P., and Kietzmann, T. C. Fixation crops and saccade coordinates for nortmann, sulewski & kietzmann (2025) (2025). https://doi.org/10.5281/zenodo.15594485.

Patterns, Volume 7

Supplemental information

Predictive remapping and allocentric coding as consequences of energy efficiency in recurrent neural network models of active vision

Thomas Nortmann, Philip Sulewski, and Tim C. Kietzmann

Supplemental methods

ALTERNATIVE ARCHITECTURES AND OBJECTIVES

To assess the robustness and specificity of our findings to the architectural and task details, we conducted two complementary analyses. First, we evaluated how architectural variations affect the emergence of energy-efficient fixation patterns and allocentric coding by testing models with different numbers of time steps per fixation and hidden layers. Second, we investigated whether similar energy efficient computational mechanisms arise under alternative training objectives by comparing our energy-minimisation approach against supervised object categorisation, temporal contrastive learning (trained for similarity with two time steps before current time step; and dissimilarity with other scene fixations), and an untrained model. These comparisons allow us to determine whether the observed phenomena are specific to energy-efficiency constraints or represent more general properties of recurrent networks processing sequential visual input. The results demonstrate that energy-efficient fixation patterns and allocentric coding are robust across architectural variants but emerge specifically from the energy-minimisation objective rather than from alternative training regimes or network architecture alone (Supplementary Fig S1).

1. ALLOCENTRIC UNIT CLUSTERING ANALYSIS

To characterise the functional organisation of allocentric coding units, we performed k-means clustering on standardised unit activations. Unit activations were first transformed using PCA (retaining the first 10 components), with the optimal cluster number (k=7) determined by silhouette score maximisation. Representative units were identified as those most correlated with cluster centroids. Seven distinct clusters emerged from the top 22 allocentric units (11 most predictive for x and y coordinates each), revealing heterogeneous spatial selectivity patterns. The majority of units (n=15) belonged to a single large cluster, while smaller specialised clusters (n=1-3) showed distinct spatial tuning characteristics. Spatial coding patterns included selectivity for upper, lower, left, and right scene regions, as well as centre/periphery coding, suggesting functional organisation into subpopulations with largely complementary spatial coverage that supports predictive remapping (Supplementary Fig S2).

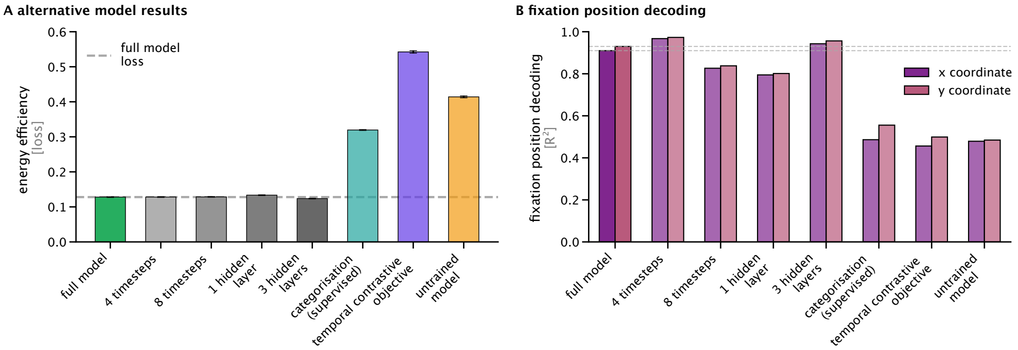

Fig. S1. Robustness of energy efficiency and allocentric coding across network architectures and objectives. (A) Energy efficiency loss comparison showing the full model and architectural variants: 4 and 8 time steps per fixation, 1 and 3 hidden layers, supervised categorisation training, temporal contrastive learning, and untrained baseline. All alternative architectures of the energy-minimisation model achieve similar efficiency (losses 0.124 to 0.134), while supervised training on the alternative objective of predicting the scene objects shows reduced energy efficiency (loss = 0.320, 99% CI: [0.318, 0.321]). Temporal contrastive learning as a scene specific temporal stability objective performs poorly (loss = 0.542, 99% CI: [0.539, 0.546]), worse than both trained models and the untrained baseline (loss = 0.414, 99% CI: [0.411, 0.417]). Error bars represent 99% confidence intervals computed across test set fixations. **(B)** Allocentric fixation position decoding performance across architectures. All alternative architectures of the energy-minimisation model successfully decode allocentric coordinates ($R^2 > 0.79$), while the supervised object detection model shows degraded allocentric coding ($R^2 = 0.49/0.56$). Temporal contrastive learning ($R^2 = 0.46/0.50$) performs similarly poorly. Both alternative objectives only allow for similar decoding as the completely untrained model ($R^2 = 0.48/0.48$). Dashed lines indicate full model performance for reference.

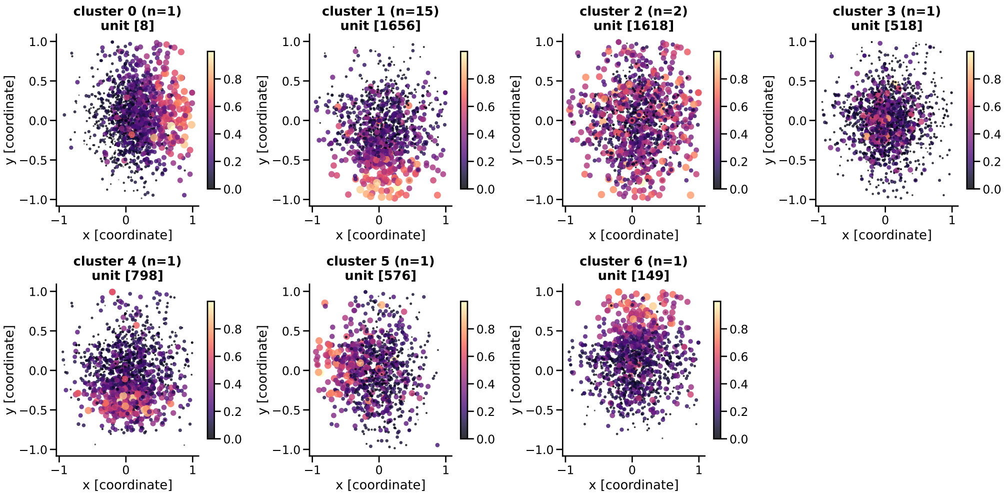

Fig. S2. Cluster representatives of allocentric coding units reveal distinct spatial tuning patterns. Spatial tuning profiles of representative units from 7 clusters identified through k-means clustering of the top 22 allocentric units (11 most predictive for x and y coordinates each). Representative units were selected as those most similar to cluster centroids. Color intensity and point size indicate normalized activation levels across spatial coordinates; magma colormap represents activation from low (dark) to high (bright yellow). Data subsampled to 4000 spatial locations for visualization. Cluster 1 contains the majority of units (n=15), while other clusters show more specialized spatial selectivity patterns. The heterogeneous tuning profiles demonstrate functional organization within the allocentric reference frame that supports predictive remapping.
